# Pediatric hypothermic submersion incident – should we do chest compressions on a beating heart?

**DOI:** 10.1186/s13049-020-00779-w

**Published:** 2020-08-20

**Authors:** Steinar Einvik, Andreas Jorstad Kruger, Sven Erik Gisvold

**Affiliations:** 1 Department of Emergency Medicine and Prehospital Services, St. Olav’s University Hospital, NO-7006 Trondheim, Norway; 2 Department of Anaesthesia and Intensive Care, St. Olav’s University Hospital, NO-7006 Trondheim, Norway; 3 Department of Research and Development, Norwegian Air Ambulance Foundation, NO-0103 Oslo, Norway; 4 Department of Circulation and Medical Imaging, Norwegian University of Science and Technology, NO-7006 Trondheim, Norway

## Abstract

**Background:**

Drowning is the third leading cause of unintentional injury death worldwide, with the highest rates of fatality among young children. To decide how to treat these patients prehospitally could be challenging in certain situations when uncertain about the adequacy of the patent’s circulation.

**Methods/case report:**

We describe a 2 year old boy surviving a 15 min hypothermic submersion in a cold river. In spite of the presence of some vital signs, we decided to do full cardiopulmonary resuscitation to the hospital. The main reason was that we were uncertain about the adequacy of the spontaneous circulation, and the transport to hospital was fairly long. The patient suffered no obvious harm and the outcome was good.

**Discussion:**

What is regarded as adequate circulation when accidentally hypothermic between 24 and 25^0^ C? A weak pulse was felt in the femoral artery with a rate of about 40–50 per minute. There were shallow, but regular respiration, and point of care ultrasound revealed a slightly dilated left ventricle and weak, but organised cardiac contractions. Despite these findings a decision was made to continue ventilations and chest compressions during helicopter transport to the University hospital.

**Conclusion:**

In an accidentally hypothermic pediatric submersion incident we decided to do full cardiopulmonary resuscitation to the hospital despite there were signs of circulation. We did no harm to the patient. Future guideline revisions should try to clarify how to handle situations with severly accidentally hypothermic patients like this, so the good outcome that is often seen in these patients could be even better.

## Introduction

Drowning is the third leading cause of unintentional injury death worldwide, with the highest rates of fatality among young children [1]. Out of hospital it can sometimes be challenging to decide how to treat these patients, but inhospital extracorporeal life support (ECLS) has revolutionized the management of hypothermic cardiac arrest, with greatly improved survival rates [2]. The reason for this is probably the neuroprotective effect of the cooling of the brain. Cerebral metabolic rate for oxygen (CMRO_2_) is reduced by approximately 8% per °C fall in temperature. At a core temperature of 28 °C the CMRO_2_ and pulse rate is 50% of the normal state [3].

## Case

We describe a 2 year old, previously healthy boy, who underwent a hypothermic submersion incident in a cold creek close to his home. The outside temperature was 6 °C. It was heavy rainfall and the small creek was like a river at the time of the incident. The boy was not observed when he fell into the river, and his father started searching for him about 10–15 min later. He was found lifeless caught in the branch of a tree 80 m downstream. The boy was evacuated and CPR was started without delay. Shortly thereafter the resuscitation was assisted by a neighbour who was a professional EMS worker.

When the first ambulance arrived on-scene 15 min later, no breathing nor pulses was detected and CPR was continued. Two air ambulance units were alarmed and dispatched. Meanwhile, assisted breathing was performed by bag, valve and mask ventilation with supplemental oxygen. An intraosseus needle was inserted and epinephrine 150 micrograms every 3 mins was administered. The estimated bodyweight was 16 kg.

The first air ambulance arrived on-scene 20 mins after the start of CPR. The crew detected still no breathing and no pulse and decided to intubate the patient with a cuffed 4.0 ETT without prior medications. The EtCO_2_ value was between 13 and 16 mmHg on-scene.

Ten to fifteen minutes later (30–35 min after start of CPR) shallow but regular respirations was observed. EtCO_2_ was still around 13–16 mmHg. A weak pulse was felt in the femoral artery with a rate of about 40–50 per minute. Then narrow-formed ECG complexes were observed on the monitor. The temperature was measured at 24.5 °C in the upper part of esophagus. Point of care ultrasound revealed a slightly dilated left ventricle and weak, but organised cardiac contractions. It was decided to stop epinephrine boluses, and despite the thready pulse that was felt in the groin it was decided to continue chest compressions in the helicopter during the 28 mins flight to St. Olav’s University Hospital in Trondheim. There were no active rewarming during transport to the hospital except the slightly raised temperature held in the cabin. The patient core body temperature was the same at arrival in the hospital as on the incident site.

The patient was transferred directly to the thoracic operation theatre and extracorporeal rewarming was prepared but never used At this time the weak femoral pulse was still palpable and chest compressions were discontinued. An arterial cannula was inserted and the blood pressure (BP) was measured at 110/70. Blood gas revealed a pH of 6.86 and S-potassium was 2.1 mmol/l. The S-Lactate was slightly above normal. At this time the body temperature measured in the upper oesophagus was 24.6^0^ C. The patient was rewarmed using 3M™ Bair Hugger. Four hours after arrival in the hospital, the core body temperature was 34.5 °C and active external rewarming was withdrawn.

The patient developed pulmonary oedema during the first hours after arrival. Nitrogenmonoksid (NO) treatment was started, and the condition improved thereafter. 15 h after arrival at the hospital the patient had normal oxygenation and a FiO_2_ at 30% with normal respirator settings.

Two days later he was weaned from the respirator, and made a full recovery with no signs of neurological injury.

## Discussion

According to the current guidelines the recommended treatment for hypothermic cardiac arrest includes CPR and transport to a hospital with extracorporeal membrane oxygenation (ECMO) capacity. A good outcome has been reported after 190 mins of CPR during transport to a hospital with extracorporeal rewarming [4]. However, if there is organised cardiac activity, one should start warming the patient externally with heated blankets and warm fluids given intravenously. The treatment protocols have highlighted the importance of treating these patients with extreme caution in order not to trigger a more malignant rhythm like ventricular fibrillation (VF) or ventricular tachycardia (VT) [4].

In this case we faced several challenges. The first was whether we should continue CPR in-flight to the hospital, despite the signs of circulation that we had found on-site. We felt a weak pulse in the groin, and there were regular, though shallow respirations. To help us decide whether the circulation was adequate, we did point of care ultrasound (POCUS) of the heart. We could see organised myocardial contractions, though the left ventricle was dilated, and not acting normal.

The second challenge was whether all these clinical findings were helpful in deciding whether the circulation in this situation should be considered adequate? In fact, the two EMS physicians on site were not certain that the cardiac contractions seen on POCUS were adequate for the child in this situation.

The third challenge was if we decided to continue chest compressions to the hospital, as we did, what was the risk for inducing VF from the patients current narrow-complex perfusing rhythm? Expert opinions [5] argue that agitation of a patient with a temperature of less than 28 °C carries a significant risk of inducing VF. On the contrary the ERC guidelines 2015 recommend to start CPR immediately if there is any doubt. We were clearly in doubt so we decided to continue CPR to the hospital [6]. The guidelines do not include decision making in situations like we encountered, which can be described as the “peri-arrest” state [5]. Despite all this, the 2015 ERC guidelines told us that when in doubt, you are not in doubt, you should start/continue CPR, and that was what we did.

The decision to continue chest compressions en-route to the hospital was based on the fact that the patient had a low core body temperature, he was not conscious, had shallow respirations and weak myocardial contractions seen on ultrasound.

The question arises whether it is correct to continue CPR during transport in a patient with a palpable pulse and spontaneous respiration. With a temperature below 25 °C the CMRO_2_ is reduced with more than 60%. A weak pulse and shallow respiration is perhaps adequate, and a “normal” adjustment to the reduced needs of the body in this situation. We had to make a quick decision on-scene and decided to do CPR assuming that oxygen transport was inadequate and needed to be supported by chest compressions and ventilations.

In this case, the patient outcome was good, so at least we did not harm the patient. There were still spontaneous circulation and respiration on arrival at hospital. Our chest compressions and intubation could easily have triggered a change of rhythm to VF or VT. If this had been the case on arrival, the patient had probably been treated with ECMO, a procedure which carries a certain risk. In the Swiss grading system (I-IV) of accidental hypothermia [7] our patient represents stage III(< 28 °C, unconscious, but with vitals present). Due to this staging system the prehospital treatment includes considering transfer to the nearest ECMO centre, and initiate ECMO treatment if signs of cardiac instability. There are published guidelines for indications for ECMO in accidental hypothermia [5]. These include:

1. Prehospital cardiac instability
2. Systolic blood pressure under 90 mmHg
3. Ventricular arrhythmias
4. Core temperature < 28 °C

Our patient had 1 and 4 above, but we could not get any measurement of the BP on-site. At arrival in the hospital the BP was measured at 110/70, as mentioned earlier. The patient was never observed with any ventricular arrhythmias.

The hospital we transported the patient to was not used to treat pediatric patients on ECMO, but are able to provide the treatment in emergencies.

Evaluation of the circulation in deeply hypothermic children is not well described in the litterature. However, in a review by Brown et al. it is stated that detecting a pulse in a hypothermic patient may be very difficult, so signs of life and pulse should be carefully checked for 60 s [4]. If no signs of life are detected, CPR should be started [4]. Moreover, they state that in patients with a temperature between 24^0^ C and 28^0^ C and vital signs present, CPR is not necessary. This is contradictory to the expert opinion mentioned earlier [5].

So the question remains; did we do anything wrong and subject the patient to unnecessary risk when treating him like he was in cardiac arrest? The question is difficult to answer. In this case the outcome was beneficial, 4 days after the incident the boy was observed walking around with no detectable neurological sequelae. The favourable outcome might be due to a short submersion time and a prompt CPR performed by witness, whom one of them was by chance a professional.. It is of course possible that the result would have been the same if CPR had not been performed. A Dutch review of pediatric hypothermic cardiac arrest in children found that good neurological outcome is more likely when return of spontaneous circulation occurs within 30 min [8]. In their material none of the 12 children receiving prolonged resuscitation(> 30 mins) after cardiac arrest due to drowning in winter had a good outcome. As such, prognostication as well as clinical decision-making might be dependent on exact timelines. The exact time our patient regained spontaneous circulation is not known.

## Conclusion

In this case report we describe a 2 year old boy surviving a 15 min hypothermic submersion in a river. In spite of the presence of some vital signs, we decided to do full CPR. The main reason was that we were uncertain about the adequacy of the spontaneous circulation, and the transport to hospital was fairly long. The patient suffered no obvious harm and the outcome was good. Future guideline revisions should try to clarify how to handle situations like this, so the good outcome that is often seen in these patients could be even better.

## Data Availability

Not applicable.

## Abbreviations

CPR: Cardiopulmonary resuscitation
ECMO: Extracorporeal membrane oxygenation
CMRO_2_: Cerebral metabolic rate of oxygene
EtCO_2_: End tidal carbondioxide
VF: Ventricular fibrillation
VT: Ventricular tachycardia
